# Genomic characterisation of multidrug-resistant Salmonella enterica serovar Kentucky ST198 isolates from various sources in Algeria, North Africa

**DOI:** 10.1099/mgen.0.001581

**Published:** 2025-11-25

**Authors:** Nabila Benamrouche, Chafika Belkader, Abdehamid Deriet-Ammar, Maria Pardos de la Gandara, Sarah Sihem Zemam, Elisabeth Njamkepo, Soraya Sadat, Amine Msela, Laëtitia Fabre, Faiza Mechouet, Dalila Torkya Boutabba, Rym Slimani, François-Xavier Weill

**Affiliations:** 1 Enterobacteria and Other Related Bacteria Laboratory, Pasteur Institute of Algeria, Algiers, Algeria; 2 Faculty of Medicine, University of Health Sciences, Algiers, Algeria; 3 Water and Food Bacteriology Laboratory, Pasteur Institute of Algeria, Algiers, Algeria; 4 Unité des Bactéries pathogènes entériques, Institut Pasteur, Université Paris Cité, Paris, France; 5 Analytical Biochemistry and Biotechnology Laboratory, University Mouloud Mammeri of Tizi Ouzou, Tizi Ouzou, Algeria; 6 Central Laboratory, El Hadi Flici Specialized Hospital Establishment, Algiers, Algeria

**Keywords:** Algeria, genomics, Kentucky, multidrug-resistant (MDR), *Salmonella*, ST198

## Abstract

*Salmonella enterica* serovar Kentucky (*S*. Kentucky) sequence type (ST) 198 has emerged as a globally disseminated multidrug-resistant (MDR) lineage posing significant public health challenges. The aim of this study was to characterise 125 *S*. Kentucky ST198 isolates collected from various sources in Algeria, including humans, animals, food and the environment, or obtained from humans in France (travellers returning from Algeria), between 2015 and 2022. Whole-genome sequencing was performed on 125 isolates to assess their genetic diversity and antimicrobial resistance (AMR) profiles. Phylogenetic analysis revealed that the Algerian *S*. Kentucky ST198 isolates were closely related to each other and belonged to the MDR lineage that emerged in Egypt before disseminating into Africa, the Middle East, Asia and Europe. These isolates also clustered closely with European and North African isolates carrying the *gyrA*_D87N mutation. We found that 90% of the isolates had MDR phenotypes, with resistance to critically important antibiotics, including ciprofloxacin, third-generation cephalosporins, azithromycin and chloramphenicol. Genomic analysis revealed that all the isolates had the three known non-synonymous resistance mutations in the quinolone-resistance-determining regions of DNA gyrase (g*yrA*) and DNA topoisomerase IV (*parC*). Multiple AMR genes were also identified, including *bla*_CTX-M-15_, *bla*_CMY-2_, *bla*_CMY-4_, *qnrB19*, *mph(A*), *cmlA1* and *floR*. The Algerian isolates also contained the main variant of SGI1, SGI1-K, with multiple rearrangements. Plasmid replicon analysis revealed that the most frequent plasmid types were IncI1 (13.6%), Col156 (8.8%) and Col(pVC) (8%). This study provides the first comprehensive genomic insight into * S*. Kentucky ST198 in Algeria, highlighting the urgent need for a reinforcement of AMR surveillance and control measures in the region. These findings enhance our overall understanding of the epidemiology and evolution of MDR *Salmonella* and highlight the importance of a One Health approach for combating the spread of resistant pathogens.

**Impact Statement**

This article provides critical insight into the genomic epidemiology and antimicrobial resistance (AMR) profiles of *Salmonella enterica* serovar Kentucky ST198, a globally emerging multidrug-resistant (MDR) pathogen. By characterising isolates from diverse sources in Algeria and from travellers returning from Algeria to France, this study documents the detection of ST198 in another North African country, suggesting regional circulation in an area with limited genomic surveillance data. Our findings indicate that the genomes in our collection belong to the ancestral ST198 lineage that emerged in Egypt before spreading across Africa, then into Europe, the Middle East and Asia and that the strains from which they were obtained are closely related to North African and European strains. All strains were fluoroquinolone-resistant, due to the acquisition of point mutations in the chromosomal *gyrA* and *parC* genes. Additional resistances, including resistance to third-generation cephalosporins (such as cefotaxime) and the last-line oral antibiotic azithromycin, were detected in diverse AMR plasmids. These findings highlight the need for enhanced One Health surveillance systems for the monitoring and control of AMR. The identification of AMR genes and plasmids in these isolates also provides valuable data for understanding resistance mechanisms and guiding public health interventions and antibiotic stewardship programmes. This work contributes to global efforts to combat AMR by bridging crucial gaps in knowledge in an understudied region and providing a basis for future research and policy-making to control the spread of this MDR lineage.

## Data Summary

The short-read sequence data generated in this study were submitted to the European Nucleotide Archive (ENA, http://www.ebi.ac.uk/ena), and their individual accession numbers are listed in Table S1 (available in the online Supplementary Material).

All the accession numbers of the publicly available sequencesused in this study are listed in Table S1.

## Introduction

Non-typhoidal *Salmonella* (NTS) is a major zoonotic pathogen causing illness in both humans and animals worldwide. Some *Salmonella enterica* subspecies and serovars have preferred reservoirs (especially poultry, pigs, cattle and reptiles). Contaminated poultry and their products are the main sources of *Salmonella* spp. infections in humans [1].

Foodborne diseases and outbreaks due to NTS are a public health concern in humans in both high- and low-income countries, causing ~93.8 million cases of foodborne illness and/or gastroenteritis and 155,000 deaths, annually [1]. Between 2018 and 2022, the European Centre for Disease Prevention and Control (ECDC) reported the occurrence of 52,690 to 91,858 human salmonellosis cases annually in Europe, with 81 deaths and more than 7,000 cases of illness resulting from more than 1,000 foodborne outbreaks in 2022 alone [2]. The US Centers for Disease Control and Prevention (CDC) has estimated that NTS cause ~150 million illnesses and 60,000 deaths globally each year [3]. However, it is difficult to evaluate the true incidence of NTS in humans and animals in developing countries due to the lack of epidemiological surveillance systems [4].

Human salmonellosis is often a mild, self-limiting disease. However, invasive infections commonly occur in children, the elderly and immunocompromised patients and are associated with an increase in the risk of severe complications and death [5,6]. In such cases, ciprofloxacin (a fluoroquinolone), cefotaxime [a third-generation cephalosporin (3GC)] and azithromycin (a macrolide) are the recommended antibiotic treatments [5,6]. Infections with antimicrobial drug-resistant *Salmonella* species are associated with higher morbidity and mortality [5,6]. The emergence and spread of resistance to ciprofloxacin, 3GCs and even carbapenems in *Salmonella* therefore constitute a serious public health concern. In 2024, the World Health Organisation (WHO) placed fluoroquinolone-resistant *Salmonella* in the high-priority pathogens group, and 3GC-resistant and carbapenem-resistant *Salmonella* in the critical-priority group, based on the immense threat that these strains pose to human health [7]. The use of antibiotics in humans and domestic livestock creates a selection pressure that may lead to the emergence of multidrug-resistant (MDR) strains, and the spread of these strains has been enhanced by the globalisation of food sales and travel [8,9].

In recent years, *S. enterica* serovar Kentucky (hereafter referred to as *S*. Kentucky) has become a common NTS serovar associated with human infection [5,6, 10]. Before the 1990s, this serovar was associated principally with poultry and was susceptible to all antibiotics. The MDR *S*. Kentucky sequence type 198 (ST198) lineage emerged in the early 1990s, due to the chromosomal integration of *Salmonella* genomic island 1 variant K (SGI1-K), a mobilisable element encoding resistance to multiple antibiotics, including aminoglycosides, beta-lactams, sulfamethoxazole and tetracycline, through the acquisition of the resistance genes *aac(3)-Id*, *aadA7*, *bla*_TEM-1_, *sul1* and *tet(A*) [8,9, 11]. Since the early 2000s, the MDR *S*. Kentucky ST198 lineage has accumulated three chromosomal mutations in the quinolone-resistance-determining regions (QRDRs) of the *gyrA* and *parC* genes, leading to high-level resistance to ciprofloxacin [CIP^R^, minimum inhibitory concentration (MIC) ≥4 mg l^−1^] [8,9, 11]. The first ST198 MDR-CIP^R^ *S*. Kentucky isolate reported was obtained in 2002, from a French tourist who had travelled to Egypt. This isolate was resistant to amoxicillin, streptomycin, spectinomycin, gentamicin, sulfamethoxazole, tetracycline and ciprofloxacin [12].

Phylogenomic studies have suggested that the MDR *S*. Kentucky ST198 lineage emerged in Egypt in the early 1990s. This lineage then became CIP^R^ and spread rapidly across Africa, the Middle East and Southern Asia before disseminating to Europe and North America. Human infections with MDR *S*. Kentucky ST198 in Europe and North America were initially predominantly associated with travel to North Africa or South-East Asia [8,14]. Some isolates subsequently became highly drug-resistant through the acquisition of resistance genes encoding an extended-spectrum beta-lactamase (CTX-M), a cephamycinase (CMY) or a carbapenemase (OXA-48, VIM and NDM), frequently associated with the IncC and IncI1 plasmids [8,14].

Poultry is the major reservoir of MDR-CIP^R^ *S*. Kentucky ST198, contributing greatly to its global spread, especially in Europe and, more recently, Asia [8,10]. This lineage is highly prevalent among poultry isolates in countries in which fluoroquinolone administration is uncontrolled in poultry production [8,14]. In the USA, *S*. Kentucky is the serovar most frequently isolated from poultry. However, the vast majority of *S*. Kentucky isolates from poultry are susceptible to antimicrobial drugs and belong to a different ST, ST152 or a single-locus variant of ST152 [15].

In Algeria, *Salmonella* infections are not considered to be notifiable diseases unless they occur in a context of foodborne illness outbreaks. Between 2017 and 2022, the Enterobacteria and Other Related Bacteria Laboratory at the Pasteur Institute of Algeria, which is responsible for the laboratory surveillance of human and non-human *Salmonella* infections, received 299 *Salmonella* isolates from humans and 322 from other sources, many from northern cities: food (*n*=193), animals (*n*=89) and environment (*n*=40). *S*. Kentucky accounted for 16.4% (49/299) of the human isolates and 21.1% (68/322) of the non-human isolates. All human *S*. Kentucky isolates and 86.8% of the non-human isolates were CIP^R^.

The population structure and antimicrobial resistance (AMR) determinants of *S*. Kentucky in Algeria have never been reported. We therefore performed a microbial genomics study on this pathogen isolated from human and non-human sources in Algeria and from travellers returning to France from Algeria.

## Methods

### Bacterial isolates collected in this study

The Enterobacteria and Other Related Bacteria Laboratory (EORB, Pasteur Institute of Algeria) received 621 *Salmonella* spp. isolates between 2017 and 2022 from hospital, veterinary and food safety laboratories. In total, 117 of these isolates were identified as *S*. Kentucky (49 from humans and 68 from non-human sources). All human isolates were obtained from sporadic cases. One isolate in this collection (IPA-Ken-19-049) was received in 2019 but was actually isolated in 2015.

Between 2016 and 2021, the French National Reference Centre for *Escherichia coli*, *Shigella* and *Salmonella* (FNRC-ESS, Institut Pasteur, Paris, France) received 52,183 clinical isolates of *Salmonella* (1 per patient), including 661 *S*. Kentucky isolates (167 in 2016, 137 in 2017, 166 in 2018, 112 in 2019, 40 in 2020 and 42 in 2021). Fifty of these *S*. Kentucky isolates were obtained from individuals reporting travel to Algeria (11 in 2016 and 2017, 15 in 2018, 10 in 2019, 3 in 2020 and none in 2021). Thirty-eight of these isolates were sequenced in the framework of national surveillance (4/11 in 2016, 10/11 in 2017, 11/15 in 2018, 10/10 in 2019 and 3/3 in 2020), whereas 12 isolates were only serotyped.

We selected 125 *S*. Kentucky isolates for this study, 80 of which were of human origin: 42 from the EORB [i.e. all 42 isolates that could be recovered from the 49 stock cultures (stored as stab cultures at ambient temperature)] and 38 from the FNRC-ESS (i.e. all the routinely sequenced isolates from the 50 patients returning from Algeria). Most of the human isolates were obtained from stools (*n*=71; 88.7%), the others being obtained from blood (*n*=3; 3.7%), ascitic fluid (*n*=2; 2.5%), bronchoalveolar lavage fluid, a pelvic fistula sample, a urine sample and an anal specimen (*n*=1; 1.2% for each).

We selected 45 of the 68 non-human *S*. Kentucky isolates from the EORB based on their source, year of isolation, recovery rate from stock cultures and budget constraints. They were obtained from food (*n*=27; merguez-type beef sausage, pastry and minced beef), animals (*n*=9; bovine stools, chicken meat, bovine carcasses and a duck) and the environment (*n*=9; seawater, water, surfaces at poultry-rearing facilities and litter).

We also performed a phylogenomic analysis including an additional 188 publicly available *S*. Kentucky ST198 genomic sequences (Table S1). These genomic sequences were obtained from isolates of various origins (human, various livestock sectors, pets, wildlife, food and the environment) from 44 countries in the Middle East, North Africa, Europe, America and Asia, between 1937 and 2022 [8,9, 14, 16]. In total, 97 of the 188 *S*. Kentucky ST198 genomes (2 historical strains, including the serotype reference strain isolated in 1937, and all 95 isolates from the MDR lineage that passed the EnteroBase quality control) were obtained in the first comprehensive phylogenetic study of this pathogen [8], 88 (all but one of all available Illumina sequences) were from a study dealing with emerging CTX-M-14b [extended-spectrum *β*-lactamase (ESBL)]-producing isolates in 8 European countries between 2009 and 2019 [9], 2 were representative genomes from a clonal population of CTX-M-14.1 (ESBL)-producing isolates in Zimbabwe (2017–2020) [14] and 1 corresponded to a CTX-M-14b-producing isolate from China, 2019 [16]. These 188 genomes included three (08-9918, 201005456 and 201301062) from human isolates collected from patients returning to France from Algeria in 2008, 2010 and 2013 [5,6, 8, 10].

### Phenotypic identification, serotyping and AMR testing

*Salmonella* isolates were identified by conventional methods (Gram staining, oxidase and the API 20E system), and serotyping was performed based on the White–Kauffmann–Le Minor scheme [17].

Antimicrobial drug susceptibility testing (AST) was performed on the 125 *S*. Kentucky isolates studied, either by the disc-diffusion method on Müller–Hinton agar in accordance with the Clinical and Laboratory Standards Institute (CLSI) guidelines [18], with a panel of 13 antimicrobial drugs (Bio-Rad, Marnes-La-Coquette, France) – ampicillin (10 µg), cefotaxime (30 µg), imipenem (10 µg), ertapenem (10 µg), gentamicin (10 µg), kanamycin (30 µg), nalidixic acid (5 µg), ciprofloxacin (5 µg), trimethoprim (5 µg), sulphonamides (300 µg), tetracycline (30 µg), chloramphenicol (30 µg) and azithromycin (15 µg) – or by the microdilution method with EUVSEC3 plates (Sensititre, Thermo Fisher Scientific, Cleveland, OH, USA). The MIC of ciprofloxacin was determined with E-test strips (bioMérieux, Marcy l’Etoile, France) or on EUVSEC3 plates. Resistance to ciprofloxacin was defined as an MIC ≥1 µg ml^−1^. Isolates displaying resistance to at least one agent in each of three or more antimicrobial categories were considered MDR [19].

### Whole-genome sequencing

Whole-genome sequencing was performed at the FNRC-ESS, on the *Plateforme de Microbiologie Mutualisée* (P2M) of the Pasteur International Bioresources network (PIBnet, Institut Pasteur, Paris, France). The MagNAPure 96 system (Roche Diagnostics, Meylan, France) was used for DNA extraction, libraries were prepared with the Nextera XT kit (Illumina, San Diego, CA, USA) and sequencing was performed with the NextSeq 500 system (Illumina) generating 150 bp paired-end reads. All reads were filtered with FqCleanER v21.10 (https://gitlab.pasteur.fr/GIPhy/fqCleanER) to eliminate adaptor sequences and discard low-quality reads with phred scores < 28 and a length <100. Assemblies were generated with SPAdes v3.15.2 [20].

The short reads from all 125 isolates from the FNRC-ESS and the EORB were uploaded to EnteroBase (https://enterobase.warwick.ac.uk/species/index/senterica) [21], and a workspace containing all these isolates can be found at https://enterobase.warwick.ac.uk/a/133549. Serovar was predicted with various tools integrated into EnteroBase: ‘Serotype (Predicted)’ in the ‘Achtman 7 Gene MLST’ tool, SISTR1 [22] and SeqSero2 [23] in the ‘Serotype(SISTR1+SeqSero2)’ tool. Multilocus sequence typing (MLST) [24] and core-genome MLST (cgMLST) [25] were performed with various tools integrated into EnteroBase.

The paired-end short reads were mapped onto the complete genome sequence of *S*. Kentucky strain 201001922 (GenBank accession no. CP028357.1) with snippy v4.6.0 (https://github.com/tseemann/snippy), using a minimum depth of 10× across the reference genome and a ratio of heterozygous single-nucleotide variants (SNVs)/homozygous SNVs <0.9. Recombination was masked with Gubbins v2.4 [26]. The resulting SNV alignment of 2,662 positions was used to create a maximum-likelihood phylogeny with RAxML v8.2.12 [27], with a GTR+G base substitution model and 1,000 bootstraps. Phylogenetic trees were visualised and annotated with Interactive Tree Of Life (iTOL) v6 (https://itol.embl.de) [28].

We searched for genetic determinants of resistance (acquired AMR genes and chromosomal point mutations) to 12 classes of antimicrobial drugs with the ResFinder v4.5.0 database from the Center for Genomic Epidemiology (http://genepi.food.dtu.dk/resfinder): aminoglycosides, *β*-lactams, colistin, oxazolidinone, fosfomycin, macrolides/lincosamides/streptogramin, phenicol, quinolones, rifampicin, sulphonamides, tetracycline and trimethoprim. Plasmid content was analysed by evaluating the plasmid replicon content of the genomes with the PlasmidFinder v2.1 database of the Centre for Genomic Epidemiology (https://cge.food.dtu.dk/services/PlasmidFinder/).

We investigated the presence and organisation of the *Salmonella* genomic island 1 (SGI1) by using each assembly as a query against the SGI1-K reference sequence of *S*. Kentucky SRC73 (accession number AY463797.8) [29] with blast v2.2.26. Contigs containing SGI1 backbone or AMR genes (as determined by ≥95% nucleotide identity and ≥95% query coverage) were considered to be part of the SGI1.

### Epidemiological data

Basic epidemiological data, including date and site of isolation and international travel (for the 80 human isolates), were recorded for all isolates (*n*=125).

### Statistical analysis

Comparisons of categorical variables were performed with chi-square (*χ*²) tests. A *P-*value below 0.05 was considered statistically significant.

## Results

### Phenotypic characterisation of the *S*. Kentucky isolates studied

The characterisations of all 125 *S*. Kentucky isolates collected in this study were confirmed by biochemical identification and classical serotyping.

All isolates were resistant to ciprofloxacin (MICs ≥2 µg ml^−1^) (Table S1). Most isolates (89.6%; 112/125) were MDR. The proportion of MDR isolates was 86.2% (69/80) for human isolates and 95.5% (43/45) for non-human isolates. This difference was statistically significant (*P*<0.05). The most frequent resistance profile was concomitant resistance to ampicillin, ciprofloxacin, sulphonamides and tetracycline (*n*=44; 35.2%). Five isolates (4.0%, 5/125), all of human origin, were resistant to 3GCs. Three of these isolates produced ESBLs and two produced AmpC *β*-lactamases. Six isolates (4.8%, 6/125) – all MDR and of human origin – were resistant to azithromycin, whereas two MDR human isolates (1.6%, 2/125) were resistant to chloramphenicol.

### MLST and cgMLST typing of the *S*. Kentucky isolates studied

We confirmed, by *in silico* serovar prediction, that all 125 isolates belonged to *S*. Kentucky [125 using ‘Serotype (predicted)’, 124 by SISTR1 (one being monophasic C2-C3:i:-) and 24 by SeqSero2 (one being monophasic 8:i:- and the remaining were classified as -:i:z6 due to the lack of an O-antigen call] and ST198. They clustered with the cgMLST HC2000_528 superlineage, and all but two belonged to HC20_528 (a very extended cluster, with more than 4,200 representative genomes in EnteroBase at the time of our most recent search on 16 September 2025). At a higher cgMLST discrimination level, 88 different HC5 clusters were observed for the Algerian isolates. The vast majority (70/88, 79.5%) of these HC5 clusters comprised only 1 isolate. Seventy of these HC5 clusters contained only isolates from the study (65 HC5 clusters with one isolate, 3 clusters with 2 isolates and 2 clusters with 3 isolates).

### Core-genome SNV-based phylogenetic analysis of the *S.* Kentucky ST198 studied

**Fig. 1.**
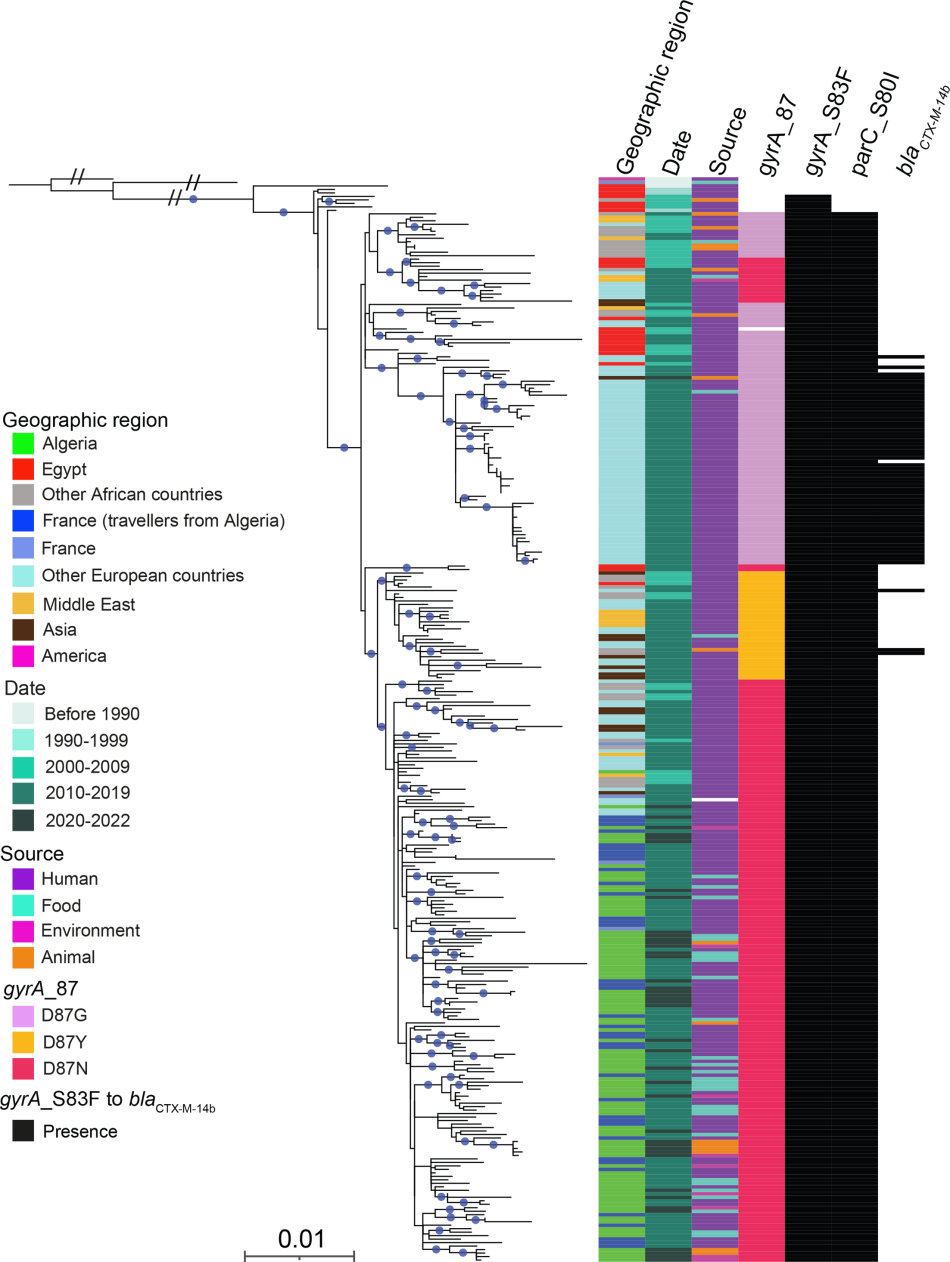
Maximum-likelihood phylogenomic tree for 311 *S*. Kentucky ST198 isolates. The tree is rooted on the *S*. Kentucky 98K reference strain. The columns on the right indicate the geographic origin, year of isolation, source and selected AMR genes (see inset legend) of the isolates. The double slash (//) indicates an artificial shortening of this branch for visualisation. Blue dots indicate bootstrap values ≥95%. The scale bar indicates the number of substitutions per variable site (SNVs).

The phylogenetic relationships between the Algerian *S*. Kentucky genomes and their position in the global phylogenetic context of *S*. Kentucky ST198 were studied by performing a maximum-likelihood phylogenetic analysis on 311 isolates (the 125 genomes studied plus 186 publicly available genomes, as 2 of the original dataset of 188 genomes, 201007297 and 201111973, were filtered out due to poor-quality sequencing) with 2,662 non-recombinant SNVs (Figs1^ 2^ and S1).

**Fig. 2.**
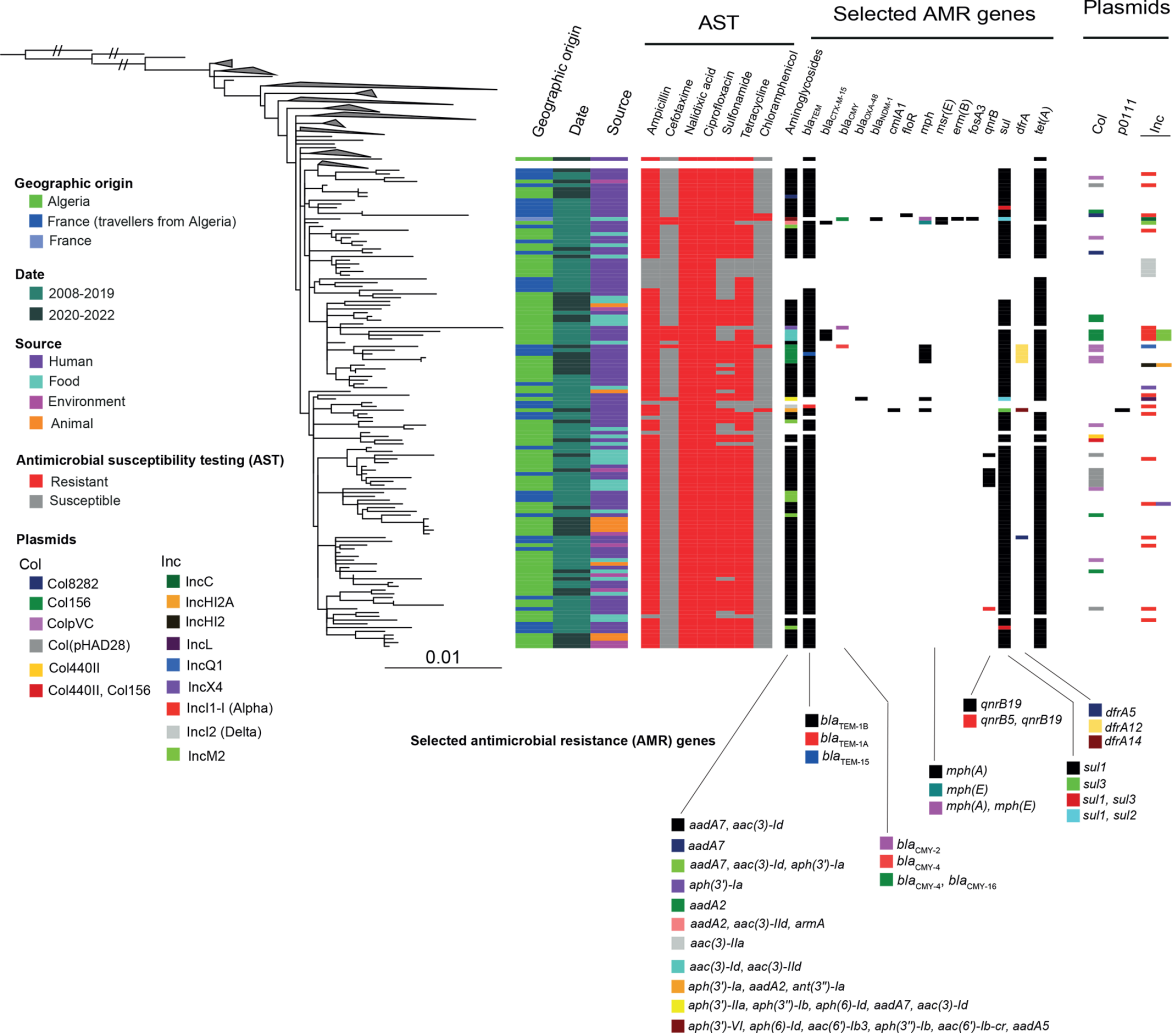
Maximum-likelihood phylogenomic tree for the *S*. Kentucky ST198 isolates from Algeria. As in Fig. 1 but with the clades not comprising the 125 studied Algerian isolates collapsed. The columns on the right indicate the geographic origin, year of isolation, source, AST results, selected AMR genes and plasmid types of the isolates. A black bar indicates presence. See the inset legends for the meaning of coloured bars. The scale bar indicates the number of substitutions per variable site (SNVs).

The 125 genomes collected in Algeria or from travellers returning from Algeria to France clustered together (mean pairwise distance of 31 core genome SNVs; minimum 1; maximum 72) within the CIP^R^-ST198 lineage, regardless of year of isolation or source (human, animal, food or environment). Three of the 186 publicly available *S*. Kentucky ST198 genomes added to the phylogeny (08-9918, 201005456 and 201301062) were recovered from travellers returning from Algeria to France in 2008, 2010 and 2013, respectively. They also grouped closely with these 125 more recent isolates from Algeria (Fig. S1). A carbapenemase (NDM-1)-producing *S*. Kentucky isolate (201401673 also named 2014LSAL00827) recovered in France in 2014 from a tank containing turkey meat [8,29] also clustered with the 128 Algerian *S*. Kentucky ST198 genomes (Table S1). The genomes closest to these 128 Algerian *S*. Kentucky genomes came from isolates obtained in Europe (France, Belgium, Spain and the Netherlands) and North Africa (Morocco).

### AMR genes and structures in the *S*. Kentucky isolates studied

Our analysis identified AMR genes encoding resistance to eight classes of antibiotics (aminoglycosides, *β*-lactams, macrolides, phenicol, quinolones, sulphonamides, tetracycline and trimethoprim) and the presence of these genes was consistent with the antimicrobial susceptibility testing data (Table S1).

All genomes contained the triple mutation [*gyrA*_Ser83Phe (S83F), *gyrA*_Asp87Asn (D87N) and *parC_*Ser80Ile (S80I)] in the QRDR of the *gyrA* and *parC* genes. In addition to these QRDR mutations, most of the genomes (*n*=104, 83.2%) also carried AMR genes against four classes of antimicrobial drugs: *β*-lactams, aminoglycosides, sulphonamides and tetracycline. Three penicillinase genes were identified: *bla*_TEM-1B_ (111 genomes, 88.8%), *bla*_TEM-1A_ (one genome, 0.8%) and *bla*_TEM-15_ (one genome, 0.8%). Three isolates contained the ESBL *bla*_CTX-M-15_ gene (2.4%) and two isolates carried AmpC genes, *bla*_CMY-2_ in one and *bla*_CMY-4_ in the other. The Algerian ST198 genomes, including those producing an ESBL, were clearly separated from the sublineage carrying the ESBL gene *bla*_CTX-M-14b_, which emerged in Europe. This emerging sublineage contained the triple mutation but with an alternative *gyrA_*D87G mutation. The vast majority of the isolates (84.8%, 106/125) harboured at least one aminoglycoside resistance gene (the cryptic chromosomal gene, *aac(6′)-Iaa*) [30], was identified in all genomes but was not taken into account). In total, 108 isolates contained between 2 and 6 AMR genes encoding resistance to aminoglycosides. The most frequent aminoglycoside resistance genes were *aac(3)-Id* (also known as *aacA5*) (*n*=100; 80%) and *aadA7* (*n*=97; 77.6%). The *sul1* (*n*=106; 84.8%) and *tet(A*) (*n*=112; 89.6%) genes were also present in most isolates. Other AMR genes were identified with a much lower prevalence, including the *dfrA* genes (*dfrA12, dfr*A5 and *dfrA14*) conferring resistance to trimethoprim in seven (5.6%) isolates. The *mph(A*) gene, conferring resistance to azithromycin, was found in six (4.8%) isolates. A plasmid-mediated quinolone resistance gene, *qnr*B19, was identified in seven genomes (5.6%). Finally, genes conferring resistance to chloramphenicol (*cmlA1* and *floR*) were identified in only two isolates (1.6%). One (201301062) of the three human isolates collected from patients returning to France from Algeria between 2008 and 2013 reported in a previous study [8] contained the *bla*_OXA-48_ and *mph(A*) genes and another (201005456) contained the *bla*_CTX-M-15_, *armA*, *mph(E*) and *msr(E*) genes.

**Fig. 3.**
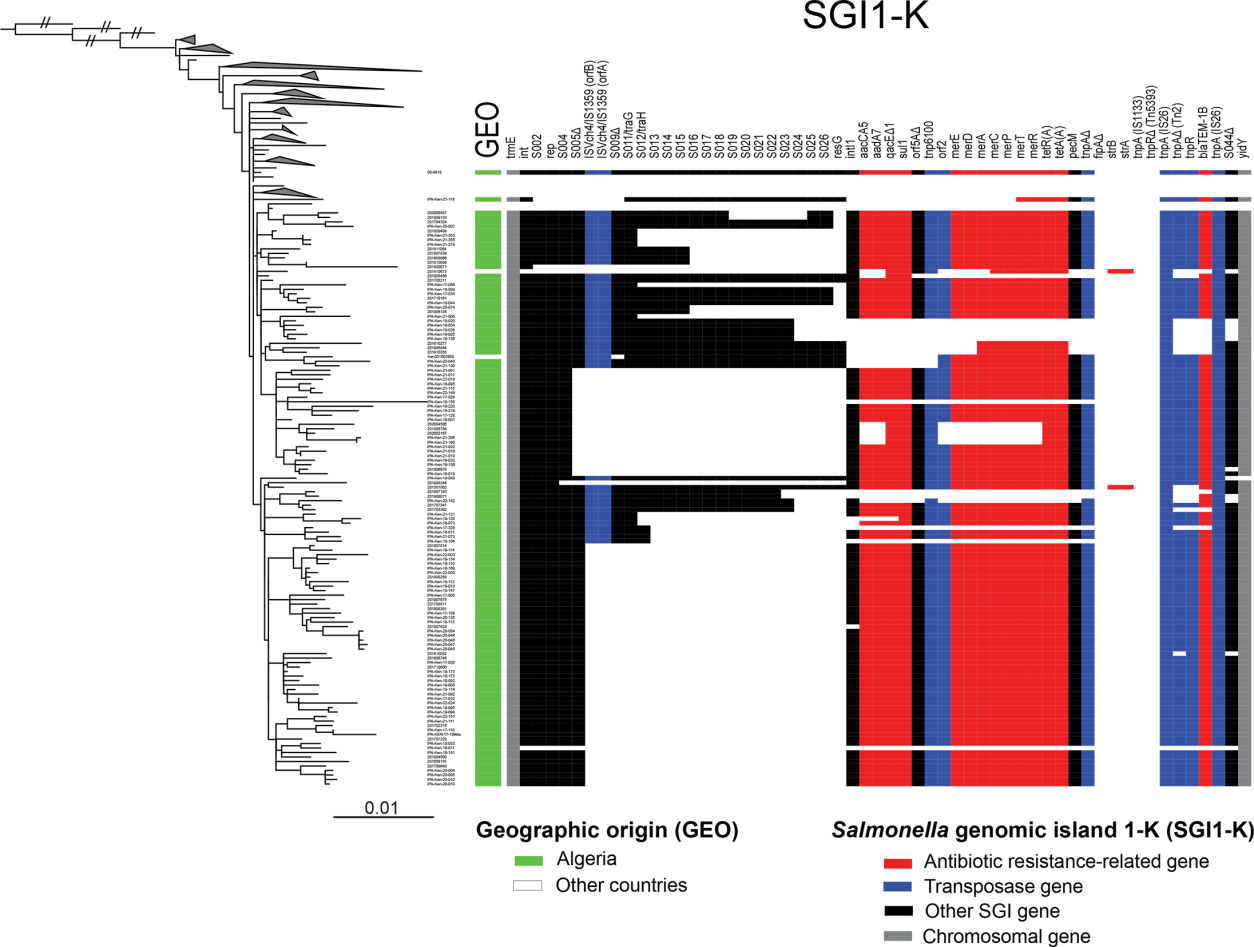
Structure and organisation of *Salmonella* genomic island 1 variant K (SGI1-K) in the *S*. Kentucky ST198 isolates from Algeria. As in Fig. 1 but with the clades not comprising the 128 Algerian isolates (125 studied and the 3, 08-9918, 201005456 and 201301062, published previously) collapsed. The columns on the right indicate the geographic origin (Algeria vs. another country) and the presence or absence of *Salmonella* genomic island 1 variant K (GenBank accession no. AY463797.8) genes. The SGI1-K genes are coloured according to category (see inset legend). Please note that this blast analysis of SGI1-K genes was not performed on long-read sequences, and we cannot, therefore, rule out the possibility that some AMR-related or transposase genes might be located on plasmids or elsewhere on the chromosome, rather than in SGI1-K. The scale bar indicates the number of substitutions per variable site (SNVs).

A blast analysis of the assemblies of the 128 *S*. Kentucky ST198 isolates from Algeria (125 from this study and the 3 previously published, 08-9918, 201005456 and 201301062) indicated the presence of an SGI1-K-like element inserted into the known chromosomal locus (i.e. between the *trmE* and *yidY* genes) in all but one of the isolates (IPA-KEN-19-011) (Fig. 3). None of the Algerian isolates had the complete SGI1-K according to the published reference sequence of *S*. Kentucky SRC73 (accession number AY463797.8, coordinates 1146-49899) [31]. In particular, Tn*5393* (containing the streptomycin resistance genes *strA* and *strB*) was not observed in our isolates. The coverage of SGI1-K was ≥90% (maximum 95%) for only 10 isolates (7.8%) and below 60% for 92 isolates (71.9%). All the Algerian isolates had various deletions encompassing AMR genes and/or SGI1-K backbone genes (Fig. 3). Many isolates (53/128, 41.4%) displayed a large deletion encompassing 20 contiguous genes of the SGI1-K backbone [from IS*1359* (also known as IS*Vch4*) to *resG*]. A second deletion involving one additional gene (from the S005 gene to the *resG gene*) was observed in 24 isolates (18.8%). These genetic rearrangements of SGI1-K were well correlated with the phylogenetic clustering of the isolates (Fig. 3).

Plasmid replicon content analysis with PlasmidFinder revealed a wide range of Inc families in the 125 *S.* Kentucky genomes studied (Table S1). Forty‑nine (39.2%) isolates (34 of human origin, 69.4%) carried at least 1 plasmid, covering 13 different replicon types. IncI1, Col156, Col(pVC), Col(pHAD28) and IncI2 were the most abundant, in 17 (13.6%), 11 (8.8%), 10 (8%), 8 (6.4%) and 5 (4%) isolates, respectively. Nine isolates (7.2%), all of human origin, harboured two or more plasmid types. No plasmid replicon was detected in 76 (60.8%) isolates, including all 9 isolates from animals.

## Discussion

Our study has a clear limitation in the relatively small number of *Salmonella* isolates received by the Pasteur Institute of Algeria and studied here, particularly for isolates of non-human origin. This small number of isolates can be explained by their collection not through targeted, systematic sampling as part of a national surveillance programme, but through passive and opportunistic sampling (as part of research collaborations, outbreak investigations or routine diagnostic activities). However, consistent with previous studies of human *S*. Kentucky ST198 in other North African countries [5,6, 8, 10, 32], all *S*. Kentucky isolates from Algeria belonged to the CIP^R^-ST198 lineage. Both human and non-human isolates collected from unrelated patients with sporadic infections, from different regions of Algeria and from different non-human sources, over a 6-year period clustered together in the global core-genome SNV analysis. The vast majority of these isolates belonged to new cgMLST HC5 clusters never before seen in EnteroBase (despite the presence of 715,705 *Salmonella* genomes, including >4,000 *S*. Kentucky ST198 with a triple QRDR mutation at the time of the search), suggesting that a particular strain of this CIP^R^-ST198 lineage had already been circulating for several years in Algeria. This epidemiological pattern contrasts with that observed in Europe, where most CIP^R^-ST198 infections are associated with travel, despite a recent increase in the number of domestic cases or cases following travel within Europe [5,6, 8, 10, 13]. The genomic clustering of isolates obtained from travellers returning from Algeria to France with isolates collected locally in Algeria is consistent with these previous reports of most European cases being linked to travel, particularly to North Africa [5,13]. Hence, 73.3% (272/371) of the 371 cases of human infection caused by CIP^R^ *S*. Kentucky in France between 2002 and 2011 were associated with travel to North Africa (Algeria, 69 cases; Egypt, 29 cases; Morocco, 147 cases; and Tunisia, 27 cases) [6].

The 125 Algerian genomes clustered with publicly available genomes from European and African countries, the Middle East and Asia, and the QRDR mutations identified (*gyrA_*S83F, *gyrA_*D87N and *parC_*S80I) were identical. Previous epidemiological investigations demonstrated that strains carrying the *gyrA_*D87N mutation spread from Egypt to North Africa, the Middle East, Europe and Asia [5,10].

A large proportion of the isolates (83.2%, 104/125) – 63 (60.6%) of human origin and 41 (39.4%) of non-human origin – carried genes conferring resistance to at least 5 antimicrobial classes. Previous studies have shown that multidrug resistance in *S*. Kentucky ST198 often results from chromosomal acquisition of the *Salmonella* genomic island SGI1, in particular the SGI1-K variant, which harbours an MDR region composed of multiple transposons (Tn*3*-like, Tn*5393*, Tn*1721* and Tn*21*) and an In*4*-type integron [8,10]. The resistance gene content of most Algerian isolates – *bla*_TEM-1_, *aacA5*, *aadA7*, *sul1* and *tet(A*), conferring resistance to ampicillin, streptomycin, gentamicin, sulfamethoxazole and tetracycline – and our analysis of the SGI1 backbone confirms that the Algerian isolates contain the main variant of SGI1, SGI1-K [8]. The presence of several transposons and insertion sequences (ISs) within SGI1 is known to cause diverse genetic rearrangements resulting in multiple variants of this element with various AMR profiles, ultimately leading to SGI1-Qs variants in which the entire MDR region is removed by IS*2*6. The *S*. Kentucky isolates containing SGI1-Qs in the absence of plasmids carrying AMR genes are generally resistant only to nalidixic acid and ciprofloxacin. At least seven of our isolates probably contain SGI1-Qs. We also confirm here that not only the MDR region but also the SGI1 backbone can also be affected by these IS- and transposon-driven genetic rearrangements [33,34]. The second most frequent deletion observed in our study (from the S005 gene to the *resG* gene) was also observed in *S*. Kentucky ST198 isolates from the SLK-2 sublineage collected from various sources in the North Asian part of Russia between 2018 and 2022 [34]. These Russian isolates also had the same triple mutation in the QRDR [i.e. with *gyrA*_Asp87Asn (D87N)] as the Algerian isolates. Unfortunately, the publication reporting these Russian isolates appeared while this manuscript was in its final stage of preparation and was not, therefore, included in our study. We cannot, therefore, currently confirm their phylogenetic relatedness. Our study of the structure of SGI1-K is clearly limited by the use of short-read contigs rather than long-read sequences. We cannot, therefore, rule out the possibility that some regions assigned to the SGI1-K here might actually be located elsewhere in the chromosome or on plasmids. We were also unable to identify additional insertions of genes and inversions of DNA regions within the SGI1-K.

The higher rate of multidrug resistance observed in non-human isolates than in clinical isolates (*P*<0.05) is consistent with previous reports highlighting the contribution of animals and food products to the spread of *S*. Kentucky ST198 [5,6, 8, 10]. Many studies have reported that poultry and poultry products – widely distributed internationally – have served as major drivers of the global dissemination of *S*. Kentucky ST198, particularly in developing countries since 2005 [5], but the limited number of poultry isolates included in this study (*n*=3) made it impossible to draw firm conclusions regarding the reservoirs of this pathogen in Algeria. However, a sampling of 14 butcheries in the province of Skikda (northeastern Algeria) during the 2014–2016 period revealed a contamination of chicken meat with *Salmonella* at 8 sites (57.1%), with CIP^R^ *S*. Kentucky accounting for 36.8% (7/19) of all the *Salmonella* isolates obtained [35]. CIP^R^ *S*. Kentucky was also isolated from laying hens and broilers in another province of Algeria (Sétif) in 2021–2022 [36]. *S*. Kentucky ST198 has also been sporadically reported in animals other than poultry and in food products not derived from poultry, including camels, horses, pigs, dogs, birds of prey, seafood and spices [5,6, 8, 10]. The identification of five isolates from cattle and cattle-derived products between 2015 and 2020 in our study is noteworthy. Between 2017 and 2021, *S*. Kentucky ST198 (Kentucky-II) was commonly associated with cattle in the USA [37]. However, the targeted sampling of a larger number of isolates would be required to confirm or rule out the possibility of bovine sources constituting an additional source of infection in Algeria.

These results enhance our knowledge of antibiotic resistance in *S*. Kentucky ST198 in the Mediterranean basin, where highly drug-resistant clinical isolates were already reported in the early 2000s. Up until 2011, studies by Le Hello and coworkers [6,10] described strains resistant to multiple antimicrobial classes, including fluoroquinolones, 3GCs and, in some cases, carbapenems and azithromycin. Carbapenemase-producing *S*. Kentucky CIP^R^-ST198 isolates were detected in Tunisia in 2009 [32] and in Morocco in 2010 [6], with resistance mediated by OXA-204 and VIM-2, respectively. In 2013, a carbapenemase (OXA-48)-producing *S*. Kentucky CIP^R^-ST198 clinical isolate (201301062, included in our dataset) was identified in France in a traveller returning from Algeria [8,32]. The *bla*_OXA-48_ gene is also widely reported in *Enterobacteriaceae* in Algeria [38,40]. However, in our collection of 125 human and non-human isolates obtained between 2015 and 2022, none of the *S*. Kentucky CIP^R^-ST198 isolates carried a carbapenemase gene or displayed phenotypic resistance to carbapenems.

One point of concern is that 4% (5/125) of the isolates carried ESBL or AmpC genes conferring resistance to 3GCs, which are recommended for the treatment of complicated or severe salmonellosis [41]. Three clinical isolates from Algeria harboured the *bla*_CTX-M-15_ gene, which is highly prevalent in this country and has been detected in various *Salmonella* serovars, such as Heidelberg, Infantis and Senftenberg, and in other *Enterobacteriaceae* isolates from humans and other sources [10,42,45]. By contrast, despite previous reports describing *bla*_CTX-M-14b_ in *S*. Kentucky CIP^R^-ST198 in Europe, southern Africa (including Zimbabwe) and Asia (China) [9,11, 14, 16], this gene was not detected in our isolates. Another matter of concern is the detection of six azithromycin-resistant isolates (4.8%) carrying *mph(A*), as azithromycin is increasingly recommended for the treatment of severe *Salmonella* infections [41].

In conclusion, this study provides the first genomic characterization of a substantial collection of *S*. Kentucky ST198 isolates from Algeria. Our findings show that isolates collected between 2015 and 2022 were all CIP^R^ and mostly MDR and, regardless of their source, were closely genetically related and different from contextual isolates. Our data are consistent with the local circulation of this high-risk lineage. Although we cannot draw any firm conclusions due to the small number of animal isolates, our data suggest that cattle and cattle-derived products may also constitute a reservoir in Algeria. The rational use of antimicrobial agents in both human and veterinary medicine, together with the strict regulation of veterinary fluoroquinolone use by Algerian authorities, is urgently needed. A One Health approach is also essential to contain the spread of MDR-CIP^R^ *S*. Kentucky ST198.

## Supplementary material

10.1099/mgen.0.001581 Uncited Fig. S1.

10.1099/mgen.0.001581 Uncited Table S1.

## Abbreviations

AMR: antimicrobial resistance
AST: antimicrobial drug susceptibility testing
cgMLST: core-genome MLST
ESBL: extended-spectrum *β*-lactamase
3GC: third-generation cephalosporin
IS: insertion sequence
MDR: multidrug-resistant
MIC: minimum inhibitory concentration
MLST: multilocus sequence typing
NTS: non-typhoidal *Salmonella*
QRDR: quinolone-resistance-determining region
*S*. Kentucky: *Salmonella enterica* serovar Kentucky
SNV: single-nucleotide variant
ST: sequence type

## References

[1] Majowicz SE, Musto J, Scallan E, Angulo FJ, Kirk M (2010). The global burden of nontyphoidal *Salmonella gastroenteritis*. Clin Infect Dis.

[2] European Food Safety Authority (EFSA), European Centre for Disease Prevention and Control (ECDC) (2023). The European Union One Health 2022 zoonoses report. EFS2.

[3] Plumb I, Fields PP, Bruce B (2024). Travel-Associated Infections & Diseases.

[4] Djeghout B, Ayachi A, Paglietti B, Langridge GC, Rubino S (2017). An Algerian perspective on non-typhoidal *Salmonella* infection. J Infect Dev Ctries.

[5] Le Hello S, Hendriksen RS, Doublet B, Fisher I, Nielsen EM (2011). International spread of an epidemic population of *Salmonella enterica* serotype Kentucky ST198 resistant to ciprofloxacin. J Infect Dis.

[6] Le Hello S, Harrois D, Bouchrif B, Sontag L, Elhani D (2013). Highly drug-resistant *Salmonella enterica* serotype Kentucky ST198-X1: a microbiological study. Lancet Infect Dis.

[7] World Health Organization (WHO) (2024). WHO Bacterial Priority Pathogens List, 2024: bacterial pathogens of public health importance to guide research, development and strategies to prevent and control antimicrobial resistance. https://www.who.int/publications/i/item/9789240093461.

[8] Hawkey J, Le Hello S, Doublet B, Granier SA, Hendriksen RS (2019). Global phylogenomics of multidrug-resistant *Salmonella enterica* serotype Kentucky ST198. Microb Genom.

[9] Coipan CE, Westrell T, Hoek A, Alm E, Kotila S (2020). Genomic epidemiology of emerging ESBL-producing *Salmonella* Kentucky bla_ctx-m-14b_ in europe. Emerg Microbes Infect.

[10] Le Hello S, Bekhit A, Granier SA, Barua H, Beutlich J (2013). The global establishment of a highly-fluoroquinolone resistant *Salmonella enterica* serotype Kentucky ST198 strain. Front Microbiol.

[11] Biggel M, Horlbog J, Nüesch-Inderbinen M, Chattaway MA, Stephan R (2022). Epidemiological links and antimicrobial resistance of clinical *Salmonella enterica* ST198 isolates: a nationwide microbial population genomic study in Switzerland. Microb Genom.

[12] Weill F-X, Bertrand S, Guesnier F, Baucheron S, Cloeckaert A (2006). Ciprofloxacin-resistant *Salmonella* Kentucky in travelers. *Emerg Infect Dis*.

[13] Westrell T, Monnet DL, Gossner C, Heuer O, Takkinen J (2014). Drug-resistant *Salmonella enterica* serotype Kentucky in Europe. Lancet Infect Dis.

[14] Mashe T, Thilliez G, Chaibva BV, Leekitcharoenphon P, Bawn M (2023). Highly drug resistant clone of *Salmonella* Kentucky ST198 in clinical infections and poultry in Zimbabwe. *NPJ Antimicrob Resist*.

[15] Tate H, Hsu C-H, Chen JC, Han J, Foley SL (2022). Genomic diversity, antimicrobial resistance, and virulence gene profiles of *Salmonella* Serovar Kentucky isolated from humans, food, and animal ceca content sources in the United States. Foodborne Pathog Dis.

[16] Lei CW, Zhang Y, Wang XC, Gao YF, Wang HN (2020). Draft genome sequence of a multidrug-resistant Salmonella enterica serotype Kentucky ST198 with chromosomal integration of bla_CTX-M-14b_ isolated from a poultry slaughterhouse in China. J Glob Antimicrob Resist.

[17] Grimont PAD, Weill FX (2007). Antigenic Formulae of the Salmonella Serovars, 9th ed.

[18] Clinical and Laboratory Standards Institute (CLSI) (2025). Performance Standards for Antimicrobial Susceptibility Testing, 33rd ed.

[19] Magiorakos A-P, Srinivasan A, Carey RB, Carmeli Y, Falagas ME (2012). Multidrug-resistant, extensively drug-resistant and pandrug-resistant bacteria: an international expert proposal for interim standard definitions for acquired resistance. Clin Microbiol Infect.

[20] Bankevich A, Nurk S, Antipov D, Gurevich AA, Dvorkin M (2012). SPAdes: a new genome assembly algorithm and its applications to single-cell sequencing. J Comput Biol.

[21] Zhou Z, Alikhan NF, Mohamed K, Fan Y, the Agama Study Group (2020). The EnteroBase user's guide, with case studies on *Salmonella* transmissions, *Yersinia pestis* phylogeny, and *Escherichia* core genomic diversity. Genome Res.

[22] Yoshida CE, Kruczkiewicz P, Laing CR, Lingohr EJ, Gannon VPJ (2016). The *Salmonella in silico* typing resource (SISTR): an open web-accessible tool for rapidly typing and subtyping draft *Salmonella* genome assemblies. PLoS One.

[23] Zhang S, den Bakker HC, Li S, Chen J, Dinsmore BA (2019). SeqSero2: rapid and improved *Salmonella* serotype determination using whole-genome sequencing data. Appl Environ Microbiol.

[24] Achtman M, Wain J, Weill F-X, Nair S, Zhou Z (2012). Multilocus sequence typing as a replacement for serotyping in *Salmonella enterica*. PLoS Pathog.

[25] Alikhan NF, Zhou Z, Sergeant MJ, Achtman M (2018). A genomic overview of the population structure of *Salmonella*. PLoS Genet.

[26] Croucher NJ, Page AJ, Connor TR, Delaney AJ, Keane JA (2015). Rapid phylogenetic analysis of large samples of recombinant bacterial whole genome sequences using Gubbins. Nucleic Acids Res.

[27] Kozlov AM, Darriba D, Flouri T, Morel B, Stamatakis A (2019). RAxML-NG: a fast, scalable and user-friendly tool for maximum likelihood phylogenetic inference. Bioinformatics.

[28] Letunic I, Bork P (2021). Interactive Tree Of Life (iTOL) v5: an online tool for phylogenetic tree display and annotation. Nucleic Acids Res.

[29] Dierikx C, Börjesson S, Perrin-Guyomard A, Haenni M, Norström M (2022). A European multicenter evaluation study to investigate the performance on commercially available selective agar plates for the detection of carbapenemase producing *Enterobacteriaceae*. J Microbiol Methods.

[30] Magnet S, Courvalin P, Lambert T (1999). Activation of the cryptic aac(6’)-Iy aminoglycoside resistance gene of *Salmonella* by a chromosomal deletion generating a transcriptional fusion. J Bacteriol.

[31] Hamidian M, Holt KE, Hall RM (2015). The complete sequence of *Salmonella* genomic island SGI1-K. J Antimicrob Chemother.

[32] Ktari S, Le Hello S, Ksibi B, Courdavault L, Mnif B (2015). Carbapenemase-producing *Salmonella enterica* serotype Kentucky ST198, North Africa. J Antimicrob Chemother.

[33] Intuy R, Supa-Amornkul S, Jaemsai B, Ruangchai W, Wiriyarat W (2024). A novel variant in *Salmonella* genomic island 1 of multidrug-resistant *Salmonella enterica* serovar Kentucky ST198. Microbiol Spectr.

[34] Kuleshov KV, Pavlova AS, Kremleva AA, Karpenko AE, Mikhaylova YV (2024). Genomic diversity and analysis of resistance determinants of *Salmonella enterica* subspecies *enterica* serotype Kentucky isolated in Russia. Zh Mikrobiol Epidemiol Immunobiol.

[35] Samia D, Bakir M, Rachid E, Chaffia B, Omar B (2021). Prevalence and genotypic characterization of *Salmonella spp*. from chicken meats marketed in the province of Skikda, Algeria. J Infect Dev Ctries.

[36] Djemaioune A, Todorović D, Novović K, Dramlić SM, Cherak Z (2025). High-level fluoroquinolone resistance and multidrug resistance in *Salmonella* spp. isolated from poultry, turkey flocks, and slaughterhouses in Algeria. Acta Vet Brno.

[37] Richards AK, Kue S, Norris CG, Shariat NW (2023). Genomic and phenotypic characterization of *Salmonella enterica* serovar Kentucky. Microb Genom.

[38] Djahmi N, Dunyach-Remy C, Pantel A, Dekhil M, Sotto A (2014). Epidemiology of carbapenemase-producing *Enterobacteriaceae* and *Acinetobacter baumannii* in Mediterranean countries. Biomed Res Int.

[39] Touati A, Mairi A (2020). Carbapenemase-producing *Enterobacterales* in Algeria: a systematic review. Microb Drug Resist.

[40] Aggoune N, Tali Maamar H, Assaous F, Guettou B, Laliam R (2018). Wide spread of OXA-48-producing *Enterobacteriaceae* in Algerian hospitals: a four years’ study. J Infect Dev Ctries.

[41] Shane AL, Mody RK, Crump JA, Tarr PI, Steiner TS (2017). 2017 Infectious Diseases Society of America clinical practice guidelines for the diagnosis and management of infectious diarrhea. Clin Infect Dis.

[42] Baba Ahmed Z, Ayad A, Mesli E, Messai Y, Bakour R (2012). 382 CTX-M-15 extended-spectrum β-lactamases in *Enterobacteriaceae* in the intensive care unit of Tlemcen Hospital, Algeria. East Mediterr Health J.

[43] Chenouf NS, Carvalho I, Messaï CR, Ruiz-Ripa L, Mama OM (2021). Extended-spectrum β-lactamase-producing *Escherichia coli* and *Klebsiella pneumoniae* from broiler liver in the center of Algeria, with detection of CTX-M-55 and B2/ST131-CTX-M-15 in *Escherichia coli*. Microb Drug Resist.

[44] Zhao WH, Hu ZQ (2013). Epidemiology and genetics of CTX-M extended-spectrum β-lactamases in Gram-negative bacteria. Crit Rev Microbiol.

[45] Djeffal S, Bakour S, Mamache B, Elgroud R, Agabou A (2017). Prevalence and clonal relationship of ESBL-producing *Salmonella* strains from humans and poultry in northeastern Algeria. BMC Vet Res.

