# Supplementary figures and images for: Genomic characterisation of multidrug-resistant Salmonella enterica serovar Kentucky ST198 isolates from various sources in Algeria, North Africa
Source: Microb Genom. 2025 Nov 25;11(11):001581. doi: 10.1099/mgen.0.001581 (PMC12646399; doi:10.1099/mgen.0.001581)

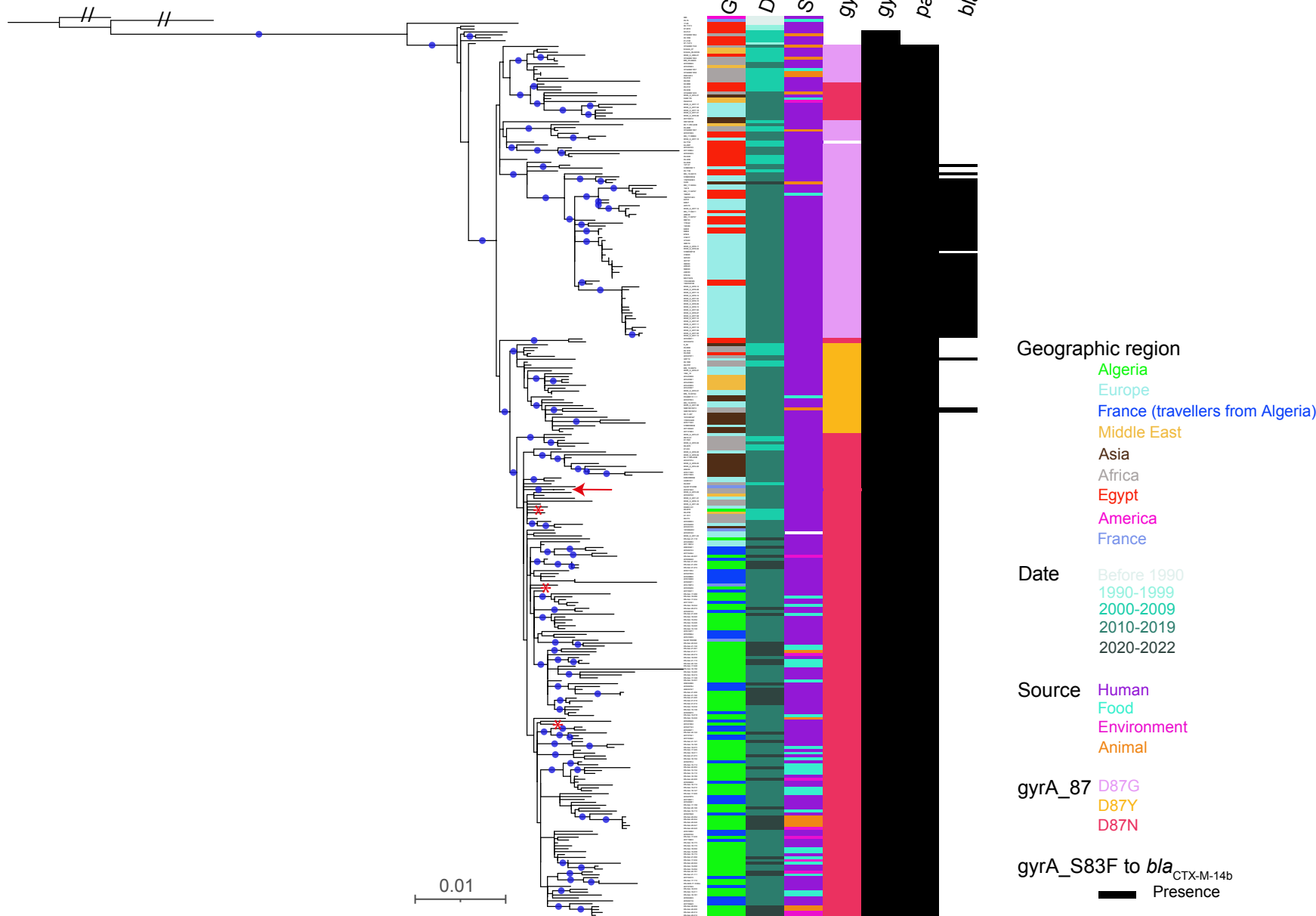

Supplement: Uncited Fig. S1. [file mgen-11-01581-s001.pdf]
